# The European influence on workers' compensation reform in the United States

**DOI:** 10.1186/1476-069X-10-103

**Published:** 2011-12-07

**Authors:** Joseph LaDou

**Affiliations:** 1 Division of Occupational and Environmental Medicine, University of California School of Medicine, San Francisco, CA 94143-0924, USA

**Keywords:** European workers' compensation, public health, physician consultation, reform, workers' compensation

## Abstract

Workers' compensation law in the United States is derived from European models of social insurance introduced in Germany and in England. These two concepts of workers' compensation are found today in the federal and state workers' compensation programs in the United States. All reform proposals in the United States are influenced by the European experience with workers' compensation. In 2006, a reform proposal termed the Public Health Model was made that would abolish the workers' compensation system, and in its place adopt a national disability insurance system for all injuries and illnesses. In the public health model, health and safety professionals would work primarily in public health agencies. The public health model eliminates the physician from any role other than that of privately consulting with the patient and offering advice solely to the patient. The Public Health Model is strongly influenced by the European success with physician consultation with industry and labor.

## Background

Workers' compensation law in the United States is derived from European social insurance. It has evolved at the federal and state levels over the past century through a long series of reform (or redesign) initiatives. There have been many hundreds of minor redesigns implemented by the state programs, the net result of which is a workers' compensation system that fails to provide the required benefits to workers. Recent reform initiatives in United States draw heavily on European workers' compensation systems, yet the European model does not offer the possibility of reform that is more than a continuation of the redesign process.

### The European Model

Workers' compensation is a European concept, dating back to German Chancellor Otto von Bismarck. By the turn of the 20^th ^century, all European countries had workers' compensation laws. The German law required employees to pay part of the costs and called for highly centralized administration. Its coverage was broad, was compulsory, and provided for nonprofit mutual employers' insurance funds [1]. Most industrialized nations now have national workers' compensation programs based on the German model [2].

The British law embodied a different approach. It was elective, administration was left to the courts, and insurance was carried through private firms. The German system was closely linked to the rest of the social insurance system. It provided for accident prevention, medical treatment, and rehabilitation, whereas the British scheme did none of these things [3]. The British system was troubled from the outset by disputes over which jobs and what industries were to be covered, resulting in the litigation that the system had been intended to replace.

### Workers' Compensation in the United States

In the United States, two separate and unequal workers' compensation systems, federal and state, function in complete separation. The federal system, under the Federal Employees' Compensation Act (FECA), was designed and operated following the German model. FECA covers 2.9 million federal employees in more than 70 different agencies along with a number of other worker groups adopted by Congress in various acts of expansion of the federal authority.

FECA provides benefits without delay, and moves disabled workers to other government programs in a non-adversarial system. It provides social insurance that most European countries would recognize as equal to their own. The FECA program operates without competition. The Secretary of Labor has exclusive jurisdiction over the entire program, including the several appeal and review processes. The Department of Labor (DOL) has few constraints on what it charges the federal agencies, passing on its costs plus, in some cases, a fee based on a pro rata share of administrative costs. Most federal agencies include workers' compensation costs in their annual appropriation requests to Congress, which makes the costs difficult to discern [4].

The United States, late in accepting workers' compensation, allowed the individual states to develop separate and unequal programs. The state laws were influenced much more by the English system than the German one. Like the original English Poor Law, prevention of poverty, not prevention of disability and its social management, was the driving concern for the development of the state workers' compensation programs [5]. The medical profession was a major opponent of compulsory social health insurance. The resulting fragmented workers' compensation arrangements now offered to most workers in state plans bear little resemblance to the federal program.

The state workers' compensation system has been confrontational with workers throughout its history, with benefits that are far from adequate. State programs pay for less than one third of the total costs of occupational injuries and illnesses, shifting most of these costs to the workers, their families, private medical insurance, and Medicare and Medicaid [6]. Ten times as many severely disabled occupational disease victims receive Social Security Disability Insurance (SSDI) or early retirement benefits as receive workers' compensation benefits [7]. Although the state programs may appear to approximate the FECA program, there are major deficiencies in coverage and benefits they offer.

Occupational injuries and illnesses, if accurately reported, would be among the five leading causes of morbidity and mortality in the United States [8]. These injuries and diseases, along with their fatalities, result in costs that are well over three times the published expenditures, and roughly 3% of the U.S. gross domestic product (GDP) [9]. Most of the costs of occupational disease are not covered by workers' compensation [10,11]. Only about one in twenty severely disabled occupational disease victims receives workers' compensation benefits. For occupational cancers, it is fewer than one in a hundred.

### Workers' Compensation in Europe

In Europe, social democracies--a mixed system that balances market forces with government assistance--have flourished since World War II. While the United States devotes only 11 percent of its GDP to redistributing income by way of social benefits, the countries of the European Union contribute more than 26 percent [12]. In Europe, there is a tradition of strong labor political organizations. With the EU's large support bases for workers' compensation, reforms are not urgent necessities there as they are elsewhere. Nonetheless, significant variations exist among EU countries. Social protection represents more than 30% of the GDP in Sweden, Denmark, France, and Germany, yet less than 15% in the Baltic countries.

The United Kingdom spends a much smaller proportion of its GDP on occupational injury and disease benefits than other European countries [13]. The new Conservative government is instituting a program of reassessing individuals receiving disability benefits. Three fourths of those evaluated in the early stages of the program have been found fit for work under new evaluation guidelines [14].

The basic criteria of the workers' compensation systems in European countries are similar. There are two distinct approaches. The first is that of the German system, with self-governed insurance associations funded by employers' contributions providing a comprehensive prevention, rehabilitation, and compensation service. In the second approach, the state administers the system for compensating occupational injuries and disease as part of its wider provision for social security and collects from employers the sums necessary to finance it. Many European countries now have mixtures of the state and private insurance systems [13].

While fewer than half of American workers are covered by short-term disability insurance, all workers in EU countries are covered against the risk of wage loss due to temporary sickness through government agencies. Wage-replacement schemes consist of social insurance covering the loss of earnings due to old age, unemployment, temporary sickness, or permanent disability. In all of the EU countries except The Netherlands, disability social security schemes are separated from compensation for occupational injuries. Coverage typically lasts up to a year, with transition to the longer-term disability insurance programs if needed. In The Netherlands, partially disabled unemployed workers are given the same benefits as totally disabled workers [15]. This offers considerable advantage over the failed attempts in the United States to deal with partial disability.

The level of compensation has a profound influence on utilization. In The Netherlands, the rate of disability in the working-age population is close to 9%, compared with an average of 6% in other European countries. Forty percent of persons with disabilities become long-term unemployed [16]. In response, comprehensive reforms have increased employer responsibilities over the past decade, and now provide a more limited benefit package. Employers are provided incentives to recruit disabled workers in order to reintegrate them into the work setting [15]. Emphasis is placed on returning workers with injury and illness to acceptable jobs, and on improving the work environment to prevent recurrence. Germany provides an example of the legally mandated role of the insurance organizations to provide specific initiatives on rehabilitation, prevention, regulation, and regulatory inspection. It is compulsory for employers to adapt the working conditions and/or to find a new work activity in the same company [17].

European countries have adopted lists of occupational diseases that are typically appended to regulatory provisions, thus ensuring the responsibility of the state. This is a concept that has never taken hold in the United States and is unlikely to be adopted. The roles of the occupational disease lists in determining compensation vary significantly. With the exception of Sweden, all EU countries have a mix between a "closed" system with a list including a certain number of diseases, and an "open" system. In Sweden there is an open system in which each claim for benefits is treated on its own merits, where all illnesses that could possibly arise from workplace exposures are considered. France has a more pragmatic closed system and lists 112 occupational diseases that require specific symptoms or pathological lesions to be present, from work that is known to cause the disease, and also specifies the time limits for compensation claims. In other EU countries, the interpretation and the use of lists are within systems that contain elements of both.

The cost of compensating occupational diseases accounts for the majority of the total costs of compensation in European countries [15]. Workers' compensation systems in Europe continue to rely on physicians and other experts to determine who receives benefits [16]. Determining the causes of occupational diseases involves a review of epidemiological and other scientific and medical evidence, and often the agreement of expert consultants as to the increased risks resulting from the occupational exposures. Significant differences exist in the established and applied diagnostic and exposure criteria in the EU countries [18]. There are differences concerning the extents to which claimants must show evidence of work exposure leading to disease. In Belgium, Italy, and Luxembourg, there is a presumption of cause. It is sufficient for victims to demonstrate that they are suffering from listed diseases and that they have been exposed to corresponding risks or that they have done jobs specified by the lists. Similarly, in France, the list of occupational diseases is considered to be a presumption of cause. In Austria, Denmark, Finland, Germany, Switzerland, Portugal, Spain, and Switzerland, the lists serve merely as guides to insurance organizations investigating the claims. Insurers will seek to establish whether a disease could have been caused by an agent on the national list while at the same time searching to find whether there are non-occupational factors that could have caused it [13].

### Reform Proposals in Europe

Reforms of workers' compensation systems are being considered in various European countries. The European Commission's Community Strategy on Health and Safety at Work proposes to significantly reduce the incidence rates of occupational injuries and illnesses by 2012. To achieve this, EU member countries must implement health and safety regulations in national legislation. Enforcement is essential, especially in small and medium-sized enterprises, where member countries must take direct measures to ensure compliance with legislation, such as inspection and the issuing of penalties [19,20].

Trade unions direct much of the discussion of reform, alleging that most occupational diseases are still ignored by the compensation systems, with under-reporting, inadequate monitoring, and a resultant lack of compensation. "Under-recognition of occupational diseases is common to all EU countries. Its most immediate consequence is a wholesale transfer of resources to the employer's benefit, with much of the cost burden being shared between victims (loss of pay as a result of reassignment or firing), and general health budgets (social security coverage of diseases, disability, and unemployment, national health system, etc.)" [21].

European countries share the concern that exclusion of people with health problems or disabilities from the labor market contributes to an increasing dependence on health-related benefits. This in turn puts pressure on the larger social protection system [15]. Even though EU member countries have developed many of the most successful workers' compensation programs in the world, the trade unions and other groups propose that substantial reforms are needed. They emphasize that

• All occupational diseases should be recognized and compensated as reliably as are occupational injuries. Procedural reform is a clear priority throughout Europe to remedy the inefficiency of the mixed system. Changes to the criteria that define occupational diseases indirectly challenge the causal presumption and should be removed from the existing schedules. Revision of schedules and recognition of new occupational diseases should be enhanced. There must be a shift in the onus of proof in light of epidemiological evidence that some occupations involve major risks of exposures to certain hazards and diseases.

• Prevention should be emphasized for chronic diseases with delayed onsets, and diseases with long latency periods such as occupational cancers. Health surveillance must be more than just medical surveillance. It must embrace surveillance of risks and exposure, the latter being particularly critical for the recognition of occupational cancers and other long-latency diseases.

• Trade union participation should be expanded so that trade unions are informed about companies with unacceptable health and safety records. In Spain, the campaigns of trade unions have led to the establishment of regional safety representatives and labor inspectors, bringing about needed improvements in occupational health and safety.

• Perceived inadequacies in levels of compensation available through social insurance combined with perceptions of injustice over employer immunity from redress under the civil law are leading to some reorientation of national "no fault" compensation systems towards a closer fit with civil law models.

• Criminal lawsuits should be reinstated where they would have salutary effects on occupational health and safety. Italy provides an example of a country where the national legal service is actively involved in identifying the occupational origins of diseases. Law officers specialized in workplace health issues work in conjunction with trade unions and victim support groups.

• The linking of occupational health and safety with public health should be enhanced to focus public health action on reducing social health inequities caused by working conditions. A public health approach to workplace health requires both political and legal changes and an extension of the spheres of preventive activities. It raises the issue of social control of the conditions of production to be consistent with both human and environmental health. It brings the concept of sustainability into the evaluation of working conditions more than ever before [22].

• Compensation systems should be integrated into a global health and safety strategy. The rapid rise in occupational injuries and diseases in developing countries cannot be ignored. The export of high-risk activities to countries in Asia, Africa, and Latin America is unacceptable. Global regulation is not nearly sufficiently developed.

Many European reforms are moving in the direction of economic incentives that reward organizations that develop and maintain safe and healthful working environments. "European workers'compensation systems always provide a combination of pure insurance functions and government regulation. The weak point of most of the compensation systems is, however, a lack of simple correlation between preventive activities and financial benefits" [23]. There are important differences in the institutional assets, in the compensation of occupational diseases, and in the kinds of incentives used in different countries regardless of the social insurance system. The fundamental difference between countries is whether the workers' compensation system is based on a competitive market between private insurance companies or on a monopoly structure, where employers cannot choose among insurance providers, Of the 27 EU countries, 19 have monopoly systems. Subsidy systems, tax incentives, and insurance-based "experience rating" are theoretically possible in all EU countries [24]. The European Agency for Safety and Health at Work (EU-OSHA) concludes that "In competitive insurance markets, effort-based incentives are more difficult to achieve. A possible solution could be the introduction of long-term contracts or the creation of a common prevention fund, financed equally by all insurers" [25].

Few countries outside Europe can attain such levels of social progress. Nonetheless, the European model is followed to varying degrees throughout the world. In many developing countries, workers' compensation is little more than a paper program where the government works in concert with industry to minimize the provision and costs of benefits [26,27]. The International Labor Organization (ILO) Convention 121 (Employment Injury Benefits Convention) is intended to ensure that the occupationally injured and diseased workers in member countries receive social security benefits that conform to the ILO's requirements for employment injury benefits and medical care and sickness benefits. To encourage countries to ratify the treaty, its requirements have a fairly low threshold. At present, only 24 of 183 member countries have ratified the treaty, including only about half of the European countries [28].

Workers' compensation reform is not widely considered outside Europe and North America. A notable exception is New Zealand, which instituted a comprehensive accident insurance system in 1974. The New Zealand no-fault system provides for compensation for all victims of injury by accident, regardless of the cause of the accident, eliminating tort remedies for such injuries [29]. Under this system, emphasis is placed on accident prevention and, when necessary, on the rehabilitation of injured persons. Tort litigation over accidents has been almost entirely eliminated by statute. Public hospitals provide medical treatment, and lump-sum awards may be granted for permanent impairment. The New Zealand system offers timely compensation to injured patients and shows evidence of effective complaint resolution and provider accountability [30].

### Reform Proposals in the United States

The legislative activity leading to the passage of the Occupational Safety and Health Act (OSHAct) raised serious questions about the fairness and adequacy of the state workers' compensation programs. Congress found the system to be in disarray, with low benefits, inadequate coverage and medical care, poor or no rehabilitation, poor administration, and excessive litigation. While the primary purpose of the OSHAct was to ensure uniformity in the application of safety and health regulations, the Act also mandated the first steps toward nationwide reform of the compensation systems. Yet, after 40 years of experience with the OSHAct, there is virtually no federal influence over the state workers' compensation programs, despite the persistence of considerable variation in administration and benefits.

### The Federal Alternative

Many reformers contend that the state workers' compensation system should be discontinued in favor of a national program with uniform coverage of health care and wage-loss benefits. There have been few calls to federalize the state workers' compensation systems in recent years. The public debate does not appear to be necessary. Most of the responsibility for compensating disabled workers already resides in the federal government, not in the state systems. The federal government not only pays for most workers' compensation benefits, it operates its own array of programs that have considerably more generous benefits than are offered by the state programs [31]. Federal funding of workers' compensation is at least four times that of state programs (See Table 1). The Social Security system is a major if not the primary source for insurance for workplace disabilities.

**Table 1:** U.S. Workers' Compensation Costs, 2008, in billions.

	State WC Programs	Federal WC Programs	Medicare/Medicaid	SSDI
Cash benefits	28.1	29.8		109.0

Health care	26.1	25.0	63.6	

Source: Sengupta I, Reno V, Burton JF, Jr: *Workers' Compensation: Benefits, Coverage*, and Costs, 2008. Washington, DC: National Academy of Social Insurance; 2010 [43]. http://www.nasi.org/research/2010/report-workers-compensation-benefits-coverage-costs-2008.

In quiet pursuit of the German model of European workers' compensation, Congress expands its authority whenever pressed by worker groups that are not well served by the state programs. The federalization of workers' compensation has been slowly unfolding, with remarkably little discussion of the costs. The Longshore and Harbor Workers Compensation Act was enacted in 1927, followed by the Black Lung Benefits Act, the Radiation Exposure Compensation Act, the War Hazards Compensation Act, and the Railroad Retirement Act. These and many other compensation programs operate with permanent positions in the federal government. The Energy Employees Occupational Illness Compensation Program Act of 2000, the newest of these programs, provides compensation for employees of the Department of Energy, its predecessor agencies, and its contractors and subcontractors who become ill as a result of the work performed in the production and testing of nuclear weapons. The Department of Labor has paid more than $6 billion in compensation and medical benefits to more than 60,000 claimants in the past nine years in just this one program [32].

### FECA Reform Proposals

The FECA program is not viewed by legislators as being in need of major reform. In that regard, its status is similar to that of European workers' compensation, undergoing revisions but not requiring major reforms. A number of reform proposals have been circulated by FECA administrators and Inspectors General [33]. These experts readily admit that FECA has serious structural problems, that it creates disincentives to return to work, and that the basic rate of FECA compensation often is more than the employee's pre-injury take-home pay. One of the proposed reform measures calls for setting disability compensation at 70% for all claimants, rather than the current varied and higher rates allowed under FECA. Another proposed FECA reform concerns the equity issue inherent in the waiting-period provision. The original purpose of a brief waiting period before benefits were awarded was to discourage claims for minor injuries and illnesses.

The most obvious problem in need of correction is that current law gives long-term FECA claimants over retirement age a choice between federal retirement system benefits and FECA benefits. Most claimants choose FECA benefits because they are more generous. A recovering FECA claimant who goes back to work risks finding that his or her retirement income will be less than if he or she had stayed on FECA benefits. Although no authority exists currently to reduce FECA benefits based on age, two types of changes have been proposed to reduce FECA benefits when employees reach an age when retirement normally occurs. One proposed change would convert FECA benefits to retirement benefits at retirement age.

### The Public Health Model

In 2006, I proposed the Public Health Model as a major departure from the European models that have dominated U.S. workers' compensation. The Public Health Model would abolish the entire workers' compensation system [34], replacing it with a comprehensive disability insurance system for all injuries and illnesses. The objective is to insure everyone equitably and to abolish the government agencies that have failed to do this over the past century. Industry and labor would deal directly with government agencies to determine a national set of benefits for injured or ill workers, with uniform incentives to return to work. Wage replacement for workers ought to be provided for a period of time stipulated by government and consistent with other social programs. The Public Health Model stipulated the following objectives:

• The current federal and state workers' compensation systems should be discontinued in favor of a national program with uniform coverage of health care and loss-of-earnings benefits. Workers in private employment should receive the same benefits as government workers.

• Resumption of tort liability should end exclusive remedy and all other provisions of the various state workers' compensation programs.

• The replacement will be a no-fault compensation system based on disability rather than cause, with an integrated approach to disability compensation such as exists in The Netherlands, where all employees are covered by a compulsory scheme that insures against loss of earnings resulting from long-term disability resulting from any injury or disease.

• There should be a national disability program similar to that in New Zealand to provide compensation for all victims of injury by accident, regardless of the cause of the accident. Disability should be defined and benefits administered without the need for health care professionals.

• Social Security (SSDI) disability benefits should be provided for all permanent injuries and illnesses. This uniform national coverage should provide an income at a level to support a dignified standard of living during disability.

• Health care should be provided by a national health care system independent of industry involvement and insurance industry control. Workers should receive the same medical care under the same conditions as all other citizens.

• Tort liability for negligence should be imposed on those who knowingly cause disability. There should be industry-wide shared liability for disability caused by or connected to industry, and society-wide shared liability for disability whose cause cannot be identified.

Europeans see a considerable advantage in the direct financing of workers' compensation by employers. European employers are better organized and have more societal power than workers' organizations, yet are still perceived to be protective of worker interests. Consequently, Europeans hold that if all health care is financed by a public budget, there will be less political pressure from employers to keep the system efficient and there could be less motivation for prevention. Employers may put less effort in better working conditions in order to avoid costs when workers' compensation becomes a societal cost. Because of these concerns, the proposed reforms in the United States are quite unlike the established European models.

The Public Health Model would impose tort liability for negligence on those who knowingly cause disability. There would be industry-wide shared liability for disability caused by or connected to industry, and society-wide shared liability for disability whose cause cannot be identified. The European view of the United States resuming tort liability is generally circumspect since workers are in a weaker legal and economic position than the employer. The U.S. experience is that workers are already in a weak position with workers' compensation and that tort liability will benefit workers. In Europe, there is political discussion of how to protect workers (whistle blowers) who report obvious bad practices by their employers. Resumption of tort liability in the United States would enhance the protections afforded whistle blowers.

Abolishing the state workers' compensation system and the federal FECA program could result in significant cost savings. The present system incurs high overhead expenses providing benefits through programs burdened with governmental bureaucracy, shared administration by private insurance companies and employers, and litigation. The public health model would treat occupational diseases in the same way all other disease is treated, removing them from the workers' compensation arena where causation must be demonstrated, often leading to litigation.

The public health model requires that we move beyond holding employers responsible for causing illness and injury through the direct costs of medical care and wage replacement. The public health model seeks to advance prevention of occupational injuries and illnesses through consultation provided by unbiased experts in health and safety. In doing so, the model adopts the European experience of physician consultation with industry [19,20,22]. France, Belgium, and Germany employ physicians to conduct inspections of worksites and examinations of employees. These physician consultants provided by government are able to mandate employer-financed occupational health services in many of the larger plants [35]. A complete hazard survey for every workplace in the country is conducted in Germany, followed by health examinations of the workers, and a plan for removal or control of hazards according to the severity of risk [3,36].

Occupational health physicians working in corporations, as well as the companies that employ them, are protected from malpractice liability by workers' compensation law. The "exclusive-remedy" provision of the law is the quid pro quo under which the employer enjoys immunity from being sued by workers for failing to be responsible for worker health in exchange for accepting financial liability for the workers' injuries. This protection would end under the pubic health model.

## Discussion

Workers' compensation law places the occupational physician in a critically important role. The physician must determine that an injury or illness is caused by work, diagnose the problem, prescribe care, and assess the extent of impairment and the ability of the worker to resume work. The common assumption is that physicians can adequately assess the extent of disability that results from occupational injury or illness. This is true only for the minor injuries that have virtually no cost impact on the workers' compensation system. When the injury is more severe, the physician's estimate of the extent of disability is far from satisfactory. Deborah Stone has pointed out that "physicians have no particular skill, training, background, or information to perform the task better than many other individuals. The failure of the physician to provide a reliable service to the worker under these circumstances results in a constant need for dispute resolution through the judicial system" [37]. Moreover, the physician's success in returning workers expeditiously to work diminishes rapidly with the increasing severity of the injury or illness.

In the public health model physicians would no longer act as gatekeepers for compensation benefits. The public health model eliminates the physician from any role other than that of privately consulting with the patient and offering advice solely to the patient. Instead, health and safety professionals would work primarily in public health agencies, enhancing the physicians' ability to represent the workers, and to approach the work setting not as employees but rather as advocates for health and safety in the workplace. If companies ignored the recommendations, regulatory agencies would intercede with appropriate enforcement. In the event that problems persisted through lack of industry compliance, the companies would be subject to litigation. Employers, without the protection of exclusive remedy, will be legally liable for their disregard of occupational health and safety. With the public health model, the costs associated consultations and prevention ought to be far less substantial than those inherent in the current system.

In many European countries, occupational medicine specialists intervene at two levels: as labor inspectors (provided by government and/or social security) and as members of the company preventive services (at one company, or in services covering many different companies). The latter are paid by employers. Inspection is carried out by State labor inspectors; risk assessment and health surveillance are carried out by company (or inter-company) physicians. A complete hazard survey for every workplace is compulsory in all the EU countries.

The public health model may now be feasible as an addition to the expanded healthcare coverage afforded by the legislation passed in 2010. A historic opportunity has been created for free choice of physicians by all injured and ill workers. Workers would receive the same health care any citizen would receive for similar injuries and illnesses, making the current system of workers' compensation health care redundant. Universal coverage can reduce total spending by eliminating the high administrative costs that are now necessary to determine eligibility for coverage [38].

European systems interpret lists of occupational diseases and compensate their victims appropriately. The United States, and a few European countries such as Finland, use a general clause or system of proof instead of a list. This requires that for a disease to be recognized as an occupational disease, a causal link to work must be proven. Occupational diseases affect 15-20% of Americans [8,39]. Conservative estimates are that 6-10% of cancers, and 5-10% of myocardial infarctions, strokes, and transient ischemia are caused by workplace factors. Occupational neurological, psychological, renal, and many other diseases are increasingly recognized [9]. Occupational diseases should be covered by workers' compensation, but their costs are largely evaded by state agencies and private insurers by amending state laws or by cost shifting. The costs of fully compensating a significant portion of heart disease, stroke, and cancer cases alone would be far beyond the current scope of workers' compensation insurance coverage. The eventual cost of an ever-expanding recognition of occupational diseases will necessitate the transfer of this burden to the mainstream of medical care where the determination of causation will no longer be necessary to treatment.

The determination of disability is increasingly viewed as a lucrative medical business. Occupational physicians have a political agenda to influence insurers to favor their opinions over those of personal physicians [40]. Some states now require proof of the physician's expertise before testimony can be admitted in court cases.

Workers' compensation medical care is much more expensive than other medical care [41,42]. Medical payments increased by 8.8% in 2008, to $29.1 billion, now for the first time accounting for over half of all workers' compensation benefits [43]. It is argued that workers' compensation medical care is delivered by physicians who provide expensive medical treatment to accelerate recovery and return to work, and that moreover, these physicians often provide information that determines income benefits, including whether an injury is compensable when a worker is ready to return to work, and assessments of permanent impairment [44].

These explanations of the extraordinary costs are not well supported, and typically come from the professional association that represents the business interests of its members. A recent survey discovered that a small group of physicians have a disproportionate effect on workers' compensation claims. These cost-intensive physicians made up 3.8% of physicians treating workers' compensation cases, but accounted for 72% of costs. They treated 16 times more claimants, and their average claim cost was four times higher than that of other physicians ($46,239 vs $11,390) [45]. Despite the increased costs, medical care provided through workers' compensation leads to poor medical results after surgery [46,47]. In the public health model, occupational physicians would not be able to exclude other physicians from providing care to their private patients.

### APHA Policy Statement

The American Public Health Association (APHA) reviewed the proposals for reform of workers' compensation, and in 2009 gave its support to many of the elements of the Public Health Model in a Policy Statement. The APHA called for increased research on work-related illness and reporting methods. A national database would lay the groundwork for research into the causes and consequences of occupational illnesses, and lead to improved diagnosis, treatment, prognosis, and ultimately, prevention of occupational diseases. There should be a comprehensive and universal reporting system for all occupational injuries and illnesses [48]. The APHA Policy Statement outlined the following objectives:

• The workers' compensation system should put prevention of injury and illness, and rehabilitation of those unable to return to work after injury and illness as its foremost goals.

• The current fragmented workers' compensation system should be replaced by a national program with uniform coverage of health care and adequate loss-of-earnings benefits for all occupational injuries and illnesses.

• The system should be a more comprehensive, no-fault compensation system based on disability, not impairment, such as exists in The Netherlands, where all employees are covered by a compulsory government administered plan that insures against loss of earnings from long-term disability resulting from any occupational injury or disease.

• The system should include a national standard of coverage for all workers, including all federal and state government workers. Individual state exemptions for seasonal agricultural workers, home care workers, domestic workers, part-time workers, contractors, immigrant workers, employees of small companies and all other special categories should be removed.

• The system should be integrated in a seamless manner with the Social Security disability program (SSDI); benefits should be provided for all permanent injuries and illnesses.

• Health care for injured workers should be provided by a national health care system independent of industry involvement and insurance industry control; health care providers should be removed from the responsibility of determining eligibility for benefits.

• The system must have mandatory root cause investigation requirements for all occupational injuries and illnesses.

• The system must have money set aside for: training of occupational health and safety professionals; preventive initiatives based on root injury and illness analyses; worker health and safety training; and mandatory reporting by health professionals

• The system should provide assistance, incentives and training in job modification and appropriate return to work.

• Where appropriate, tort and criminal liability for negligence should be permitted for those who knowingly or recklessly cause disability.

• There should be a national medical and statistical database on worker injuries, worker illnesses, worker toxic exposures and resultant diseases.

## Conclusion

European workers' compensation systems offer a number of important examples for redesign of workers' compensation in the United States. These examples are most useful to the FECA program for federal employees. The EU's far more generous support for workers' compensation results in many redesign initiatives that are not easily translated to the more serious need for reform that exists in the United States at the state level. The Public Health Model proposed in 2006 would abolish the workers' compensation system, and in its place adopt a national disability insurance system for all injuries and illnesses. Although a marked departure from European workers' compensation, the Public Health Model would embrace the European success with physician consultation with industry and labor.

## Abbreviations

DOL: US Department of Labor; EU: European Union; FECA: Federal Employees' Compensation Act; SSDI: Social Security Disability Insurance.

## Competing interests

The author has no conflict of interests. He has not accepted payment from any organization or institution. The work is not supported by any grant or institution.

## Authors' contributions

JL is the sole author of the manuscript.
